# Functional correlates of executive dysfunction in primary progressive aphasia: a systematic review

**DOI:** 10.3389/fnagi.2024.1448214

**Published:** 2024-10-18

**Authors:** Kristin Thomsen, Stefanie Keulen, Seçkin Arslan

**Affiliations:** ^1^Université Côte d'Azur, CNRS, BCL, Nice, France; ^2^Brussels Centre for Language Studies (BCLS), Vrije Universiteit Brussel (VUB), Brussels, Belgium; ^3^Center for Research in Cognitive Neuroscience (CRCN), ULB Neuroscience Institute (UNI), Université Libre de Bruxelles, Brussels, Belgium

**Keywords:** primary progressive aphasia, executive functions, neuropsychology, neurophysiological imaging, MEG, PET, fMRI

## Abstract

**Introduction:**

Recent research has recognized executive dysfunction as another component affected in Primary Progressive Aphasia (PPA). This systematic review aimed to examine what information distinctive neurophysiological markers can provide in the evaluation of executive function (EF) deficits in PPA, and to what effect executive function deficits can be assessed through the characteristics of functional markers.

**Methods:**

We conducted a systematic literature search following the PRISMA guidelines across studies that employed neuropsychological assessments and neurophysiological imaging techniques (EEG, MEG; PET, SPECT, fMRI, fNIRS) to investigate executive dysfunction correlates in PPA.

**Results:**

Findings from nine articles including a total number of 111 individuals with PPA met our inclusion criteria and were synthesized. Although research on the neural correlates of EF deficits is scarce, MEG studies revealed widespread oscillatory slowing, with increased delta and decreased alpha power, where alterations in alpha, theta, and beta activities were significant predictors of executive function deficits. PET findings demonstrated significant correlations between executive dysfunction and hypometabolism in frontal brain regions. fMRI results indicated elevated homotopic connectivity in PPA patients, with a broader and more anterior distribution of abnormal hippocampal connections of which were associated with reduced executive performance.

**Conclusion:**

Our study provides indirect support for the assumption regarding the significance of the frontal regions and inferior frontal junction in executive control and demonstrates that neurophysiological tools can be a useful aid to further investigate clinical-neurophysiological correlations in PPA.

## 1 Introduction

Primary progressive aphasia (PPA) is a neurodegenerative condition characterized by the presence of a focal and progressive dementia that impacts the language network. PPA is a form of aphasia, an acquired language disorder that impairs the ability to understand and produce language and, consequently, to effectively communicate in daily living. Unlike post-stroke aphasia, PPA is progressive, meaning that language abilities impacted by the aphasic syndrome worsen in time as the disease progresses. The aetiological mechanisms underlying PPA often involve cortical and/or subcortical atrophy caused by Alzheimer’s disease (AD) type amyloidopathy, frontotemporal tauopathy, TDP-43 pathology, or *α*-synucleinopathy (usually found in Parkinson’s or Lewy body diseases). PPA is often characterized by clinical and neuropathological similarities to AD or frontotemporal lobe dementias; however, by contrast, aphasia is the most prominent and first occurring syndrome in PPA, as the degeneration particularly impacts the left hemisphere language network, including the perisylvian frontotemporal and parietal areas (Gorno-Tempini et al., 2011; Mesulam et al., 2009; Mesulam, 2001, 2003). Atrophy in these areas leads to language impairments commonly observed in aphasia, including difficulties with word retrieval, word finding, naming, repeating, and producing syntactically complex sentences (Gorno-Tempini et al., 2011). While a gradual decline in language abilities characterizes PPA, other cognitive functions are largely preserved in the initial stages of the disorder (Gorno-Tempini et al., 2011; Mesulam et al., 2009; Mesulam, 2001). According to the latest consensus formalized in 2011, there are currently three clinical variants of PPA diagnostically differentiated based on core linguistic, cognitive, and pathological profiles (Gorno-Tempini et al., 2011; see Table 1). A nonfluent/agrammatic variant (nfvPPA), a semantic variant (svPPA), and a logopenic variant (lvPPA). Although nfvPPA and svPPA often are associated with pathologies within the spectrum of frontotemporal lobar degeneration (FTLD; Mesulam et al., 2008; Snowden et al., 2007; Spinelli et al., 2017), and lvPPA more commonly with Alzheimer’s disease (AD) pathology (Grossman, 2010; Harris and Jones, 2014), a direct correspondence between pathology and clinical manifestations is not necessarily warranted.

**Table 1:** The clinical diagnostic criteria for PPA variants (Modified from Gorno-Tempini et al., 2011).

	Nonfluent variant PPA (NfvPPA)	Semantic variant PPA (SvPPA)	Logopenic variant PPA (LvPPA)
Core symptoms	Agrammatism in language production and/or effortful, nonfluent speech and apraxia of speech	Impaired confrontation naming and impaired single-word comprehension	Impaired single-word retrieval in spontaneous speech and naming and impaired repetition of sentences and phrases
Additional possible features	Impaired comprehension of syntactically complex sentences, spared single-word comprehension, spared object knowledge	Impaired object knowledge—particularly for low-frequency or low-familiarity items, surface dyslexia or dysgraphia, spared repetition, spared speech production (grammar and motor speech)	Speech (phonological) errors in spontaneous speech and naming, spared single-word comprehension and object knowledge, spared motor speech, absence of agrammatism
Imaging-supported atrophy patterns	Predominant left posterior fronto-insular atrophy on MRI and/or predominant left posterior fronto-insular hypoperfusion or hypometabolism on SPECT or PET	Predominant anterior temporal lobe atrophy and/or predominant anterior temporal hypoperfusion or hypometabolism on SPECT and PET	Predominant left posterior perisylvian or parietal atrophy on MRI and/or predominant left posterior perisylvian or parietal hypoperfusion or hypometabolism on SPECT or PET

### 1.1 Executive functions in PPA

While language impairment is the leading characteristic of PPA’s clinical profile, recent research has provided insight into another critical aspect of cognitive decline in individuals with PPA: the deterioration of executive functions (EFs; see also Coemans et al., 2022). Executive functioning refers to several higher-level cognitive abilities that regulate and control the brain’s lower-level cognitive operations to direct behavior toward an intended goal (Alvarez and Emory, 2006; Diamond, 2013; Miyake and Friedman, 2012). Engagement with these functions, also often referred to as executive or cognitive control, requires significant cognitive effort as it involves overriding automatic responses and behaviors. Neuroanatomically, EFs are mostly mediated by the prefrontal cortices and frontal regions, and deficits in these neural networks have been found to have significant implications for EF performance (Kramer et al., 2007; Friedman and Robbins, 2022). Executive processes have also been linked to structures beyond these frontal regions, such as frontoparietal networks (Reineberg et al., 2015), and to both anterior and posterior structures (Bettcher et al., 2016).

A range of models have been proposed to clarify the disposition of EFs. Therein, the ‘unity and diversity’ model established by Miyake and colleagues distinguishes three core EF components; (a) ‘Shifting between tasks or mental sets’ (henceforth shifting), (b) ‘Updating and monitoring of working memory representations’ (henceforth updating), and (c) ‘Inhibition of prepotent responses’ (henceforth inhibition; Miyake et al., 2000, pp. 55–57). According to the model, ‘unity’ refers to the idea that EFs are not entirely independent of each other but instead share some inherent similarities. ‘Diversity’ refers to the idea that each function holds its unique features—a concept shown through differential performance contributions on cognitive tasks. Hence, overall, the unity and diversity model posits how the three core EFs described are related but still separable.

As mentioned, non-linguistic cognitive domains have been assumed to remain intact in the initial phases of PPA, leading to a pervasive dismissal of these variables in research and diagnostic procedures (Mesulam, 2001). However, recent studies have found evidence for EF deficits in all three variants of PPA (Basaglia-Pappas et al., 2023; Coemans et al., 2022; Kamath et al., 2019). Therein, the reported data show impairments in all three EF components. For example, using Trail Making Test part B and Stroop Color-Word Test to assess shifting and inhibition, Chen et al. (2018) found svPPA patients to have impaired performance in both tasks compared to healthy controls. Within this study, svPPA patients with predominant atrophy in the left hemisphere further exhibited greater impairments on the Stroop test than those with right-hemisphere variant of svPPA. Inhibition, assessed with the Stroop task, has also been impaired in lvPPA patients (Matias-Guiu et al., 2019) and nfvPPA patients (Gajardo-Vidal et al., 2024).

Following the framework of Miyake and colleagues, Coemans et al. (2022) recently conducted a meta-analysis to systematically investigate measures of EFs in PPA. The authors first evaluated and categorized commonly used EF tasks based on their functional component classification. They found that many frequently reported EF tests are limited in their association with EFs due to their dependencies on a multifaceted range of cognitive abilities. Therein, task applicability was determined by the task characteristics’ alignment with the principles proposed by the unity and diversity model. Tasks reported to align with the Miyake model are provided in Table 2. Overall, their study demonstrated poorer performance of PPA patients as a group on the EFs composite (standing for the three components) but also for inhibition and shifting separately. However, they could only include effect sizes for ‘updating’ for six studies, making it the more underreported component, and this could possibly explain why the effects of this component did not always reach significance. PPA variant was a significant moderator of the effects found for EF composite analysis, with performance of lvPPA and nfvPPA being worse than that of svPPA. The authors linked these differences to the degeneration patterns and underlying neuropathologies that can be attested for the PPA variants. Disease duration was identified as a moderator for the overall EFs composite, as well as for shifting and inhibition separately. ‘Patient age at assessment’ was a significant moderator for the overall EFs composite as well as for shifting, but not for inhibition, suggesting that older patients with PPA performed more poorly on EF tests. Interestingly, task modality (i.e., verbal/non-verbal) was not found to influence the observed effects of executive performance deficits. This discovery, showing that task modality exerts no moderating effect on EF deficits in PPA is noteworthy, as it delineates these deficits as being inherent to EFs rather than a secondary outcome of language impairments. Additionally, it suggests that EF impairments can be reliably assessed through various task modalities, thereby supporting the robustness of these findings across conditions.

**Table 2:** Classification of tasks according to EF components (Modified from Coemans et al., 2022).

Shifting	Updating	Inhibition
The plus-minus task (Jersild, 1927) The number-letter task (Rogers and Monsell, 1995) The local–global task (Navon, 1977) Wisconsin card sorting test (Berg, 1948) Trail Making Test Part B (Reitan, 1955)	The keep track task (Yntema, 1963) The letter memory task (Morris and Jones, 1990) The n-back task (Jonides and Smith, 1997) The tone monitoring task (Larson et al., 1988) The operation span task (Turner and Engle, 1989)	The antisaccade task (Hallett, 1978) The stop-signal task (Logan, 1994) The Stroop task (Stroop, 1935) The Tower of Hanoi (Arnett et al., 1997) The Flanker task (Eriksen and Eriksen, 1974) The Attention Network Test (Fan et al., 2002) Go/No Go task (Gordon and Caramazza, 1982)

Moreover, two other meta-analyses further investigated measures of neuropsychological functions in PPA (Kamath et al., 2019, 2020). Kamath et al. (2019) comprehensively compared patients with nfvPPA, svPPA, and behavioral variant frontotemporal dementia (bv-FTD). The authors found similar effect sizes for executive dysfunction in nfvPPA and bv-FTD patients, highlighting a commonality in these variants’ impairments. However, svPPA patients exhibited a notably lower effect size for executive function deficits, indicating a distinction in cognitive profiles (Kamath et al., 2019). These findings are consistent with those of Coemans et al. (2022), who found comparable EF performance deficits in lvPPA and nfvPPA patients, which were further significantly worse than those of patients with svPPA. Kamath et al. (2020), having focused on lvPPA, observed that deficits in visual set-shifting were as conspicuous as language impairments in lvPPA patients. However, due to a limited number of applicable studies, a comprehensive assessment of other EF subdomains or direct comparisons with the other PPA variants were not possible within this study, emphasizing the need for further research on the cognitive intricacies of different PPA variants and their corresponding EF profiles.

### 1.2 Functional neuroimaging and neurophysiological techniques

Existing diagnostic criteria for PPA (Gorno-Tempini et al., 2011) are grounded in behavioral profiles. In the disorder’s early stages, these clinical manifestations can overlap between variants, making it difficult to differentiate them (Gorno-Tempini et al., 2008; Mesulam et al., 2014). However, emerging data shows a potential for variant differentiation by incorporating functional imaging and physiological techniques. In general, functional neuroimaging and related techniques attempt to measure neuronal activity. The applied techniques measure resting-state/baseline levels of brain activity or the changes in brain function that occur when actively engaging in specific tasks, allowing for the investigation of regions responsible for cognitive processes. However, the way these techniques are employed varies. Within PPA research, techniques such as magnetoencephalography (MEG) are employed to monitor changes in magnetic fields related to shifts in neurophysiological responses in the brain. Ranasinghe et al. (2014) and Shah-Basak et al. (2019) conducted studies focusing on the power spectra of neural oscillations in specific frequency ranges in PPA patients. Oscillatory bands refer to distinct frequency ranges of brain wave activity, each associated with various aspects of neural processing. These bands include delta (<4 Hz), theta (4–8 Hz), alpha (8–12 Hz), beta (12–30 Hz), gamma (30–80 Hz), and high gamma (>80 Hz). Their findings revealed a notable slowing in neural oscillatory activity, particularly characterized by increased delta and decreased alpha power, which not only served as a unique predictor for PPA but also significantly correlated with the severity of executive function deficits, as indicated by changes in alpha, theta, and beta activity.

Largely, neurodegenerative dementias exhibit specific disease-related metabolic reduction patterns (Ishii, 2014). Positron Emission Tomography (PET) is a neuroimaging tool that visualizes brain metabolic processes and pathologies by detecting gamma rays from radioactively labeled tracers. Hypometabolism, characterized by a decline in metabolic rate or activity in the brain, is indicated by reduced glucose uptake and utilization in specific brain regions, suggesting a decrease in neuronal activity or dysfunction in these areas. Within the scope of PPA research, Raczka et al. (2010) identified a significant correlation between executive dysfunction and hypometabolism in various frontomedian and left frontolateral regions, as well as the left insula, left globus pallidus, caudate, and thalamus. Complementing these findings, Desgranges et al. (2007) observed hypometabolism, correlating with moderate deficits in executive function abilities and behavioral changes in svPPA patients, predominantly in the orbitofrontal areas. Similarly, Schroeter et al. (2012) reported that executive deficits in PPA were primarily linked with prefrontal hypometabolism. Collectively, emphasizing a reliance on the frontal brain structures in PPA for executive control functions.

Functional Magnetic Resonance Imaging (fMRI) measures brain activity by observing changes in the brain’s vascular hemodynamic responses as an indirect measure of neuronal activity (Logothetis, 2008). In a study by Tao et al. (2022), patients with PPA exhibited significantly higher homotopic connectivity values. This suggests that there is an increased synchronized activity between homotopic regions, that is, structurally similar areas located symmetrically in opposite hemispheres of the brain. Initially, the PPA group demonstrated a more extensive and anteriorly distributed pattern of abnormal hyperconnectivity. This means that abnormal brain synchronization was not only higher, but also spread over a larger area – specifically, toward anterior brain regions. A pattern which indicates that the disruption in brain functions affects a range of brain regions, particularly those located toward frontal areas. For the significantly abnormal connections, there was a notable correlation between elevated functional connectivity (FC) and diminished executive performance, in this case, evidenced by prolonged reaction times in the Trail Making Test. This suggests that maintained EF task performance in PPA patients is associated with homotopic FC levels more akin to those observed in the control group.

### 1.3 The current review

Summarizing the available literature shows that, in contrast to the common belief, patients with PPA exhibit significant declines in EFs compared to healthy controls across EF composites (Coemans et al., 2022; Kamath et al., 2020). Notably, the extent of these deficits varies with PPA variant and disease duration (Basaglia-Pappas et al., 2023; Coemans et al., 2022; Kamath et al., 2019). Additionally, the impact of these deficits remains consistent regardless of task modality (Coemans et al., 2022), highlighting the need for further research to enhance diagnostic and treatment approaches in clinical practice. Given that PPA is a relatively rare neurocognitive condition with large individual differences, understanding the broader context is essential. Hence, this systematic review consolidates studies investigating EF dysfunction in PPA using electrophysiological and functional neuroimaging techniques to draw conclusions on functional markers of EF dysfunction in PPA.

Given the profound personal impact and clinical importance of EFs, the emerging evidence of their impairment in PPA, and the current fragmentation of functional neuroimaging findings, the objectives for this review are (1) to examine what information distinctive functional markers can provide in the evaluation of executive function deficits in PPA, and (2) to what effect executive function deficits can be assessed through the characteristics of functional markers.

## 2 Methods

### 2.1 Identification of articles and search strategies

This systematic review followed the Preferred Reporting for Systematic Reviews and Meta-analyses guidelines (PRISMA; Moher et al., 2009). Studies from the online publication databases PubMed and Web of Science were searched for literature relevant to the review, with the last search conducted in November 2023. Each title and abstract were scanned, and relevant articles reporting on functional neuroimaging in PPA were retrieved. Only full-text articles published in peer-reviewed journals in English were retained. The following search string was utilized for the systematic review in the databases PubMed and Web of Science:

(((Primary progressive aphas*) OR (PPA) OR (progressive aphasia) OR (semantic dementia) OR (semantic variant) OR (logopenic variant) OR (agrammatic variant) OR (nonfluent variant) OR (progressive nonfluent aphasia) OR (PNFA)) AND ((EEG) OR (electrophysiological) OR (MEG) OR (magnetoencephalography) OR (PET) OR (positron emission tomography) OR (fMRI) OR (SPECT) OR (single-photon emission computerised tomography) OR (fNIRS) OR (functional near-infrared spectroscopy)) AND ((executive function*) OR (executive control) OR (neuropsych*) OR (inhibit*) OR (shift*) OR (updat*)))

**Figure 1:**
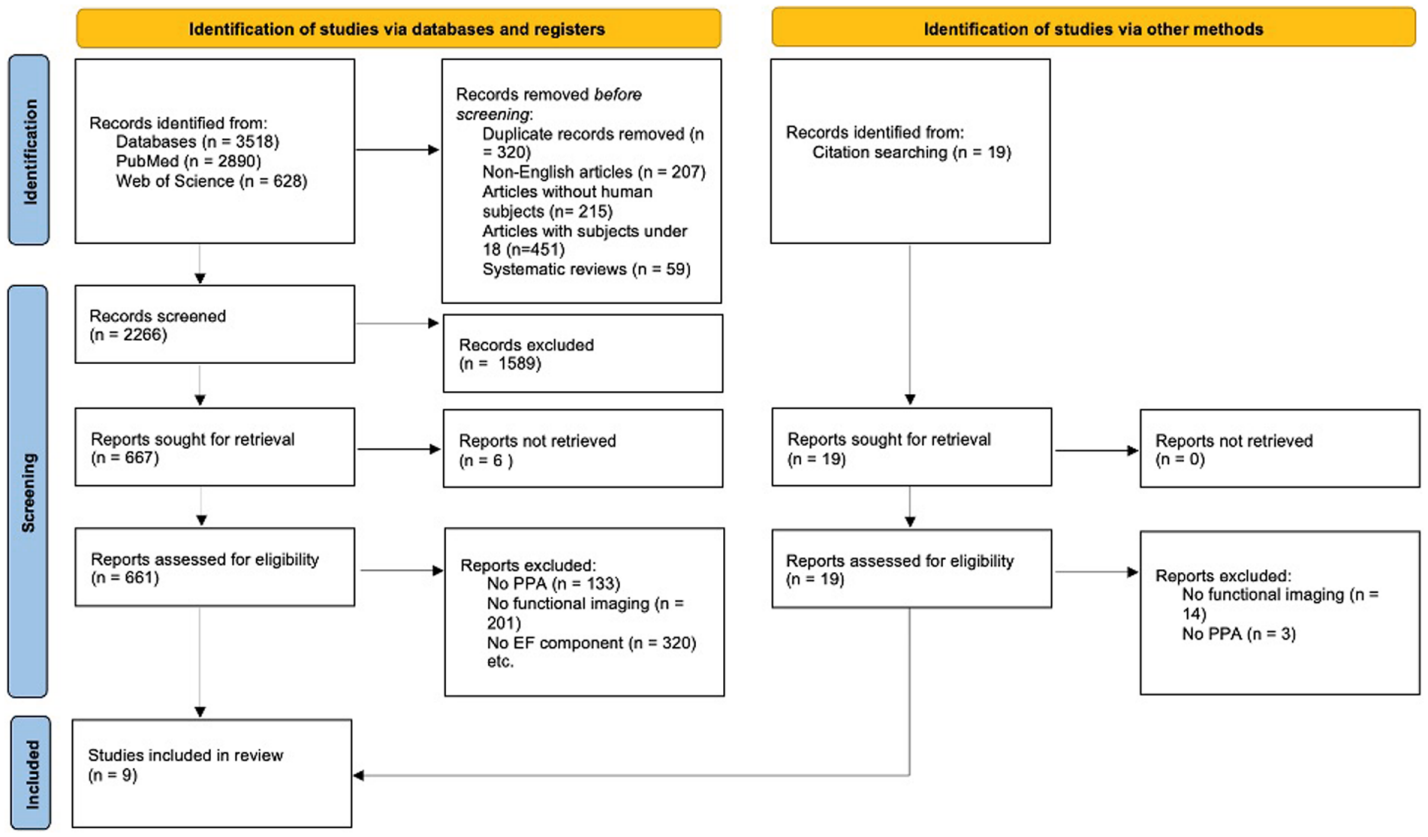
PRISMA flow diagram illustrating the process of study selection for the systematic review. The diagram outlines the number of records identified, included, and excluded at each review stage.

This search revealed 3,518 articles. Additionally, individual references were examined to obtain further relevant studies that were not accessible through the online search, equating to a total of 3,537 articles. Primary inclusion criteria for further analysis were (1) a PPA diagnosis, which was open to various diagnostic criteria and did not adhere to any one specific, standardized set of guidelines; (2) the inclusion of neuropsychological testing including the assessment of EF components, and (3) the reported use of one of the following functional assessments: electroencephalography (EEG), magnetoencephalography (MEG), positron emission tomography (PET), single-photon emission computerized tomography (SPECT), functional magnetic resonance imaging (fMRI) or functional near-infrared spectroscopy (fNIRS), see Figure 1 for the PRISMA flow diagram.

### 2.2 Data extraction

Baseline information was collected from each study; this included publication year and patient characteristics, namely, sex, age at examination, disease duration when provided, and employed diagnostic criteria for PPA; subtype is provided when identified. Additionally, the methodological approaches for each study were extracted. This included data on neuropsychological assessments, data analysis, and neurophysiological data.

## 3 Results

### 3.1 Study search and characteristics

The study characteristics from all nine studies are provided in Table 3. Hundred and twelve patients were included in the review (59 nfvPPA, 23 svPPA, 11 lvPPA, and 18 unidentified/mixed variants). Five studies included nfvPPA patients (Cooke et al., 2003; Mandelli et al., 2018; Shah-Basak et al., 2019; Tao et al., 2022), three studies included lvPPA patients (Ranasinghe et al., 2014; Schroeter et al., 2012; Shah-Basak et al., 2019), three studies included svPPA patients (Desgranges et al., 2007; Raczka et al., 2010; Schroeter et al., 2012), and two studies included undefined-PPA patients (Schroeter et al., 2012; Utianski et al., 2019). Six of the included studies employed the Gorno-Tempini et al. (2011) classification of PPA and its variants (Mandelli et al., 2018; Ranasinghe et al., 2014; Schroeter et al., 2012; Shah-Basak et al., 2019; Tao et al., 2022; Utianski et al., 2019), while four studied followed the Neary et al. (1998) diagnostic criteria for frontotemporal dementia (FTD) and its subtypes (Cooke et al., 2003; Desgranges et al., 2007; Raczka et al., 2010; Schroeter et al., 2012). The sex distribution was 51.8% female and 48.2% male, with two studies not disclosing this information. The age range of patients at examination in the included studies varied, ranging from 63.2 to 77.0 years per study, with the mean age across all included studies being 67.4 years. The mean duration of the disease post-onset was 3.4 years, ranging from 1.3 to 5.4 years; one study did not disclose this information (Cooke et al., 2003). Seven studies included age-matched or senior controls (Cooke et al., 2003; Desgranges et al., 2007; Mandelli et al., 2018; Raczka et al., 2010; Ranasinghe et al., 2014; Shah-Basak et al., 2019; Tao et al., 2022), while one study additionally included healthy younger controls (Shah-Basak et al., 2019). One study used neuropsychological data from Mayo’s Older Americans Normative Studies (MOANS) as a reference in place of a control group, providing age- and education-adjusted standard scores to assess patient performance (Utianski et al., 2019). In another study, the control group included individuals with subjective memory complaints that were not confirmed by neuropsychological and clinical evaluations. The subjects exhibited normal neuropsychological performance with only expected age-related cognitive decline. This control group was also utilized to avoid ethical concerns associated with using healthy subjects in [18F] fluorodeoxyglucose-PET studies due to radiation exposure (Schroeter et al., 2012).

**Table 3:** Study characteristics.

	Study	Imaging	Sample	Mean age at examination (years)	Disease duration (years)	Control group	Diagnostic criteria
1	Cooke et al. (2003)	fMRI	3 PNFA	77.0		11 healthy seniors	Neary et al. (1998)
2	Desgranges et al. (2007)	PET	10 SD	65.7	3.3 ± 2.5	Cognitive assessment: 21 age-matched controls Neuroimaging: 17 control subjects from a database	Neary et al. (1998)
3	Mandelli et al. (2018)	fMRI	20 nfvPPA	68.8 (7.3)	3.57 + 1.43	20 Age-matched controls	Gorno-Tempini et al. (2011)
4	Raczka et al. (2010)	PET	SD 7, FTD 7, FTLD 3			9 senior controls	Clinical and Neuropathological Criteria for Frontotemporal Dementia (1994) and Neary et al. (1998)
5	Ranasinghe et al. (2014)	MEG	5 lvPPA	63.2	5.4 + 3.3	15 Age-matched controls	Gorno-Tempini et al. (2011)
6	Schroeter et al. (2012)	PET	FTLD (13 FTD, 6 SD, 4 mixed type) 1 lvPPA	FTLD = 61.1, Other dementias = 64.9	FTLD 3.35 Other 3.77	Subjective cognitive impairment as a control group	Neary et al. (1998) and Gorno-Tempini et al. (2011)
7	Shah-Basak et al. (2019)	MEG	6 nfvPPA 6 lvPPA	69.7	1.3 + 1.2	18 young controls, 24 senior controls	Gorno-Tempini et al. (2011)
8	Tao et al. (2022)	fMRI	30 nfvPPA	68.8	3.66 + 2.42	41 senior controls	Gorno-Tempini et al. (2011)
9	Utianski et al. (2019)	PET	15 PPA-U	67.1	2.9	Mayo’s Older Americans Normative Studies (MOANS) scores	Gorno-Tempini et al. (2011)

### 3.2 EF measures

Several measures of EFs were used. Therein, studies utilized a combination of tests or basic neuropsychological batteries. The most common tests were the Trail making test-B or Modified trails (TMT-B; Reitan, 1955; Desgranges et al., 2007; Mandelli et al., 2018; Raczka et al., 2010; Ranasinghe et al., 2014; Shah-Basak et al., 2019; Tao et al., 2022; Utianski et al., 2019), and the Stroop test (Stroop, 1935; see Cooke et al., 2003; Desgranges et al., 2007; Raczka et al., 2010; Schroeter et al., 2012; Utianski et al., 2019), used to test shifting and inhibition, respectively. Other tests included the Running span tasks (Desgranges et al., 2007; Ranasinghe et al., 2014; Shah-Basak et al., 2019) and verbal fluency tasks, tests that arguably offer only a restricted assessment of EFs based on the theoretical framework of Miyake et al. (2000).

### 3.3 Neural correlates of EF measures

Ranasinghe et al. (2014) and Shah-Basak et al. (2019) investigated variations in the power spectra of neural oscillations within specific frequency ranges through MEG. They found that patients with PPA exhibited widespread oscillatory slowing. Notably, this slowing in oscillatory activity, especially the increase in delta and decrease in alpha power, was identified as a unique predictor for PPA. Furthermore, abnormalities or changes in the alpha, theta, and beta activity significantly predicted the severity level (EF scores) of executive function deficits in individuals with PPA. A summary of all findings is presented in Table 4.

**Table 4:** Details of the studies included in the systematic review and their outcomes.

	Study and Year	Imaging	Test(s) of EF	Test outcomes	Associated function(s)/region(s)
1	Cooke et al. (2003)	fMRI	TMT-B, the Stroop test	Trails B % accuracy PPA: 93.3 (8.3) CG: 98 (3.0) Stroop % accuracy PPA: 100 (0) CG: 98.6 (2.2)	There were no direct correlations between EF measure and neural activity in PPA patients; however, there was an additional recruitment of medial frontal regions.
2	Desgranges et al. (2007)	PET	TMT-B, the Stroop test, the digit running span tasks	TMT-B rt. PPA: 225.88 (99.7) CG: 133.47 (65.5) Stroop accuracy PPA: 33.3 (9.1) CG: 48.28 (6.9) Running Span accuracy PPA: 4.67 (2.1) CG: 7.33 (4.1)	Hypometabolism focused on the orbitofrontal areas, which also showed extended structural atrophy, consistent with a moderate deficit in EF abilities.
3	Mandelli et al. (2018)	fMRI	Rule Violation (RV) errors from the Delis-Kaplan Executive Function System.	Modified Trails (lines /sec) PPA: 3.6 (1.4) CG: 0.6 (0.2)	A significant negative correlation between the average number of RV errors and the functional connectivity of the newly recruited hub in the right opercular part of the inferior frontal gyrus (opIFG) with the left opIFG.
4	Raczka et al. (2010)	PET	Modified Stroop, Tower of Toronto, BADS	ED score SD: 1.8 ± 1.3 FTD: 2.8 ± 0.4 FTLD: 1.5 ± 2.1 CG: n.a.	Executive dysfunction was significantly correlated with glucose hypometabolism in several frontomedian areas (anterior cingulate and mid cingulate gyrus, anterior medial frontal cortex), left frontolateral regions (frontopolar cortex, inferior, middle and superior frontal gyri), left insula, left globus pallidus, caudatum and thalamus.
5	Ranasinghe et al. (2014)	MEG	Modified trials, the digit running span tasks	Modified Trails (lines/s) PPA: 0.15 ± 0.1 Digits span backward: PPA: 2.5 ± 0.6	Resting-state functional connectivity reductions in the left dlPFC correlated to performance deficits.
6	Schroeter et al. (2012)	PET	The Stroop test, Category fluency, BADS	Stroop task (s) PPA: 4.6 ± 2.9 CG: 1.3 ± 0.3 Category Fluency PPA: 10.1 ± 7.8 CG: 23.0 ± 2.2 Zoo Map Test 1 PPA: 5.0 ± 3.7 CG: 6.7 ± 2.5 Zoo Map Test 2 PPA: 7.6 ± 1.3 CG: 8.0 ± 0.0 Key search PPA: 6.7 ± 3.9 CG: 14.3 ± 2.4	Impairment in behavioral performance related to Stroop interference correlated with hypometabolism in the left inferior frontal junction and posterior superior and middle frontal gyrus. All executive tests were related to hypometabolism in the left inferior frontal junction.
7	Shah-Basak et al. (2019)	MEG	TMT-A + B, the digit running span tasks, MoCa	TMT-A + B n/a The digit running span tasks n/a MoCa (30) PPA: 19.5 ± 6.3 CG: 26.5 ± 1.9	Widespread oscillatory slowing associated with additional volumetric reductions in the brain. Power changes in the alpha, theta, and beta activity significantly predicted EF scores.
8	Tao et al. (2022)	fMRI	TMT-A + B	Trail Making Test A (time in second) PPA: 55.7 (21.59) 35.8 (11.9) Trail Making Test B (time in second, max 300) PPA: 166.84 (89.45) 81.2 (38.5)	Higher homotopic functional connectivity (HC) values correlated with lower EF performance. Abnormal HC connections were more widespread and anterior.
9	Utianski et al. (2019)	PET	TMT-B, MoCa	TMT-A 6.87 TMT-B 6.0 MoCa 21.07	Positive loadings in the bilateral inferior frontal lobes, left sensorimotor region, and right cerebellum and contrasted by negative loadings in all other regions, including the medial and lateral temporal lobes and lateral parietal.

BADS,Behavioural Assessment of the Dysexecutive Syndrome; CG, control group; ED score, Executive dysfunction score; FTD, frontotemporal dementia; FTLD, frontotemporal lobar degeneration; MoCa, Montreal Cognitive Assessment; SD, semantic dementia; TMT-A, Trail making test part A; TMT-B, Trail making test part B.

The PET studies found hypometabolism consistent with a moderate deficit in EF abilities in SD patients to be concentrated explicitly in the orbitofrontal areas (Desgranges et al., 2007). This hypometabolism was more extensive than grey matter loss in the temporal lobes. Moreover, significant correlations between specific brain regions and principal components were found when looking into the neurodegenerative patterns of PPA-U (Utianski et al., 2019). Therein, principal components were based on EF test scores measured by the Trail Making Test Part B (TMT-B). The second principal component showed a positive correlation between the component and activity in the bilateral inferior frontal lobes, left sensorimotor region, and right cerebellum, contrasted by negative correlations in medial and lateral temporal lobes and lateral parietal lobes. The third principal component showed a positive correlation in the left lateral and medial temporal lobes, indicating asymmetry with minimal right hemisphere involvement, and negative correlations in sensorimotor and lateral parietal regions. Furthermore, significant links were drawn between executive dysfunction and hypometabolism in various frontomedian areas (including the anterior cingulate and mid cingulate gyrus, anterior medial frontal cortex), left frontolateral regions (such as the frontopolar cortex, inferior, middle, and superior frontal gyri), left insula, left globus pallidus, caudate, and thalamus (Raczka et al., 2010). This was further associated with executive dysfunction with hypometabolism in the left frontolateral cortices, encompassing the inferior frontal junction area, previously found to be involved with cognitive control (Jourdan Moser et al., 2009). In Schroeter et al. (2012) executive deficits were also predominantly associated with prefrontal hypometabolism. Overall, the inferior frontal junctions emerged as the most consistently significant regions across various EF tasks, highlighting executive control’s reliance on frontal brain structures.

EF measures of executive dysfunction explored through fMRI show that PPA patients have elevated homotopic values (Tao et al., 2022), meaning that the PPA group showed more synchronized activity across homotopic regions. At the baseline, the distribution of abnormal HC connections was more widespread and anterior in the PPA group. For the significantly abnormal connections, elevated FC values correlated with lower executive performance, in this case, through longer Trail reaction times, showing that better task performance was associated with homotopic FC levels closer to that of the control group. The study further included a calculation of structural damage to examine its relation to FC abnormalities as a global Grey matter volume (GMV) measure. This analysis showed that abnormal homotopic FC levels showed behavioral correlations consistent with their anatomical distribution - therein, medial frontal homotopic abnormalities were associated with EF performance in PPA. Mandelli et al. (2018) revealed a significant negative correlation between task performance and the FC between the right and left opercular inferior frontal gyrus (opIFG). Higher FC was linked to fewer errors, indicating better EF performance. No significant correlation was found with volume loss or nodal metrics within these nodes. Not all fMRI studies showed significant correlations between PPA and executive function deficits. One study only found significant EF impairments for a group of five non-aphasic patients with dysexecutive and social impairments (EXEC; Cooke et al., 2003); however, additional recruitment of medial frontal regions was found in the nfvPPA variant.

### 3.4 Methodological approaches

#### 3.4.1 Data acquisition and processing

Two studies employed MEG to record neural oscillatory activity (Ranasinghe et al., 2014; Shah-Basak et al., 2019) while simultaneously incorporating T1-weighted MRI scans for MEG source modeling. MEG source analyses were conducted by computing power spectral densities using the multitaper method with a focus on delta, theta, alpha, and beta frequency bands (Shah-Basak et al., 2019) and functional connectivity analyses focusing on the alpha frequency band of specific data epochs (Ranasinghe et al., 2014).

Four studies utilized fluorodeoxyglucose PET (FDG-PET; Desgranges et al., 2007; Raczka et al., 2010; Schroeter et al., 2012; Utianski et al., 2019). Some controlled for environmental influences by minimizing external stimuli and body movement in their methodological approach (Desgranges et al., 2007; Utianski et al., 2019), while the other PET studies did not report on this (Raczka et al., 2010; Schroeter et al., 2012). The typical doses of 18FDG utilized ranged from 367 to 576 MBq (Raczka et al., 2010; Schroeter et al., 2012; Utianski et al., 2019), while one study used a smaller dose of 111 to 185 MBq (Desgranges et al., 2007). In the studies, dynamic scans were conducted, with each study adopting different protocols for the timing of image acquisition post-injection, predominantly a 10-min after a 30–50-min uptake period. Statistical Parametric Mapping for voxel-based analysis (Raczka et al., 2010; Schroeter et al., 2012) and 3-dimensional stereotactic surface projections were employed to assess hypometabolism (Utianski et al., 2019).

Three studies utilized fMRI using a 3 T scanner (Cooke et al., 2003; Mandelli et al., 2018; Tao et al., 2022). Functional connectivity (FC) was calculated using pre-processed time-series data. FC estimation included Pearson’s correlation and Fisher’s *z*-transform, with the functional connectomes subsequently averaged.

#### 3.4.2 Statistical analysis

Statistical analyses included using Pearson’s correlation and FDR correction (Ranasinghe et al., 2014), ANOVAs and post-hoc t-tests with Holm’s correction for group comparisons (Shah-Basak et al., 2019), Spearman tests and ROI analysis (Desgranges et al., 2007), general linear models with significance determined by Gaussian fields theory (Raczka et al., 2010; Schroeter et al., 2012), PCA and Spearman’s method (Utianski et al., 2019), and linear regression, and FDR adjustment, while also exploring the impact on behavioral scores with regression analyses (Tao et al., 2022).

## 4 Discussion

The clinical profile of PPA and its subtypes is predominately focused on isolated deficits in language domains. In diagnostic guidelines, the loss of speech and other language symptoms are the main causes of impaired daily functioning in individuals with PPA, while other cognitive abilities such as EFs have been described to remain relatively intact in the early stages of the disorder (Mesulam, 2001; Mesulam et al., 2009; Gorno-Tempini et al., 2011). Thus, currently, the executive profile of PPA is not considered under the consensual diagnostic criteria (i.e., Gorno-Tempini et al., 2011) and has rarely been a primary focus of research in this population. However, though language abilities remain the most salient aspect of impairment, executive dysfunction related to all variants of PPA has been reported (see Coemans et al., 2022), even in the early stages. The present systematic review aimed to examine what information functional markers provide in the evaluation of EF deficits in PPA and to what effect EF deficits can be assessed through the characteristics of functional markers. While the existing literature on neurophysiological imaging modalities concerning PPA is scarce, studies examining the neural correlates of executive dysfunction were identified for three neurophysiological techniques, MEG, PET, and fMRI. Overall, the findings of this review add to the argument of affected EFs in individuals diagnosed with PPA, further challenging the prevailing assumption of mostly unaffected EFs in this population. In the following sections, the relationship between EF deficits in PPA and their neural correlates will be discussed, focusing on the EF components shifting, updating, and inhibition and how these deficits are reflected through changes in neural activity patterns across distinct brain regions.

### 4.1 Neural correlates associated with EF deficits

A summary of EF impairments in PPA and associated neural correlates can be found in Table 5. Several brain regions were linked to EF deficits in individuals with PPA, including the orbitofrontal areas, frontomedian areas, left frontolateral regions, left insula, left globus pallidus, caudate, and thalamus. These findings suggest that specific neural networks are implicated in EFs, with significant overlap in regions traditionally associated with language processing, particularly in the frontal and temporal lobes. This indicates that EF deficits in PPA may not be entirely separable from language impairments, as both components rely on interconnected neural connections. Therein, the extent of executive dysfunction varies across PPA variants, including nfvPPA, svPPA, lvPPA, and U-PPA, suggesting variant-specific patterns of neural degeneration that impact both language and EFs.

**Table 5:** Summary of EF Components and Findings.

EF component	Key findings	Neural correlates
Shifting	Impaired in PPA	Frontal lobes, particularly the dlPFC and the IFJ, indicate a disruption in neural networks facilitating the shifting component. Increased delta power and decreased alpha power indicate neural slowing. TMT-B; Modified trails.
Inhibition	Impaired in PPA	The ACC and orbitofrontal areas show significant metabolic and structural changes. Oscillatory slowing, particularly in the theta frequency band. Stroop Test
Updating	Little data; underrepresented	This underrepresentation makes it difficult to associate any neural correlates with this component

### 4.2 Alignment of EF deficits in PPA with the unity and diversity model

The findings from 111 PPA patients reveal significant impairments in EFs when compared to healthy controls across all three EF composites conceptualized by Miyake et al. (2000). However, to what extent these deficits can be directly attributed to executive impairments or are secondary to language impairments remains a critical question. While multiple studies have included tests for the neural correlates of ‘shifting’ and ‘inhibition’, the component ‘updating’ is relatively underrepresented in existing research. This discrepancy could be due to the inherent challenges in isolating updating processes from other cognitive functions, such as working memory, limiting our comprehension of how PPA affects the spectrum of EFs in variants of PPA. Primarily, studies applied the TMT-B and Stroop Test, which, according to the Miyake et al. (2000) framework, aligns with the shifting and inhibition components of EFs. Therefore, the relative underrepresentation of the updating component in PPA demonstrates the necessity for further investigation into the component by employing tasks that more accurately measure these distinct processes. Moreover, The Digit Span Backwards and Running Span tasks have frequently been used to test updating. However, as these tasks focus more strongly on working memory capacities rather than the continuous processes of renewing and replacing information, they do not effectively align with the principles of the Unity and Diversity model. This misalignment shows a critical need for incorporating different tasks that can more accurately reflect the updating component, such as the n-back task, which directly challenges the ability to continuously update information. Nonetheless, it is important to note that research is currently limited for all three EF components.

### 4.3 Neurophysiological insights into PPA’s executive deficits

In the current review, MEG studies, detecting changes in brain activity with high temporal accuracy, highlighted the presence of a widespread oscillatory slowing in PPA. Therein, EF abilities were impaired, and the severity of these deficits was associated with an increase in delta, theta and alpha power, and decreased beta power, providing a predictor for executive dysfunction through oscillatory abnormalities. Nonetheless, the processes underlying these abnormal oscillatory activities are not well understood, indicating a need for further research. PET imaging revealed hypometabolism in brain regions associated with executive control, such as the frontomedian and left frontolateral regions (Desgranges et al., 2007; Raczka et al., 2010). This hypometabolism, or reduced metabolic activity was further correlated with executive dysfunction. The role of the frontomedial and frontolateral regions, specifically the anterior cingulate cortex and the inferior frontal junction, is commonly associated with cognitive control and EF processes (Brass et al., 2005; Ridderinkhof et al., 2004). In individuals with AD, PET scans have shown a reduced uptake of glucose in certain brain regions as a reflection of decreased neural activity (Camandola and Mattson, 2017; Hoyer, 1985) and worsening hypometabolism has been linked to both progressive cognitive and functional decline (Landau et al., 2011). Additionally, the spatial resolution of fMRI demonstrates abnormal functional connectivity patterns, particularly increased synchronized activity across homotopic regions, which correlated with reduced executive performance (Tao et al., 2022). Abnormalities or changes in homotopic connectivity can be indicative of alterations in functional processes, in the association with neurological or neurodegenerative conditions, or the outcomes of brain injury. Considering these modalities, electrophysiological techniques such as electroencephalography (EEG) could offer additional insights into the neural mechanisms underlying EF deficits in PPA. This is because the high temporal resolution of EEG combined with its accessibility makes it a suitable method for examining the neural dynamics associated with executive dysfunction. Furthermore, a multimodal imaging approach, combining neurophysiological imaging such as MEG, PET, fMRI, or EEG with structural imaging, would offer a more comprehensive understanding of cognitive impairments in PPA.

### 4.4 Methodological considerations

As a reflection of the rarity of PPA and its variants, the sample sizes in all the reviewed studies were relatively small. This limitation makes it challenging to generalize findings across the PPA variants. PPA typically presents heterogeneity in symptoms; thus, a single-case approach could offer a profound understanding of the relationship between EF deficits and neural correlates by allowing for a more detailed examination of individual differences, thereby providing insights that might be obscured in larger studies. Nonetheless, group studies allowing for the implementation of advanced statistical methods to facilitate comparisons across PPA variants would still optimally surpass single-case approaches by enhancing the generalizability and robustness of findings. Another promising methodological approach identified in the reviewed papers includes PCA analysis, which captures the variability in EF performance and neurophysiological imaging within a small cohort of patients.

### 4.5 Limitations

This review is not without any limitations. Firstly, as the literature on the neural correlates of executive dysfunction is scarce, only nine studies met the inclusion criteria for the systematic review. Within these studies, the assessment of EFs was based on correlations between EF task performance and neural correlates; no direct task-based activity was available. Secondly, there is a lack of consensus on what task domains unequivocally can be part of executive functioning. There is currently no established standard for defining and evaluating EFs, leading to variability in research findings and task selection across clinical assessments. This review only included studies that used tasks in line with the characteristics of EF components defined by Miyake et al. (2000). However, it is common for studies to report tasks as being indicative of executive functions, though the criteria for how and/or whether these tasks relate to executive processes remain unclear. Therein, it has been argued that given that EFs are not a uniform concept, there has been a lack of a clear standard measure in research, which is essential for comparing and evaluating potential EF measures. A limitation of this review is the underrepresentation of the updating component of executive functions, with most studies focusing on shifting and inhibition, which limits a comprehensive understanding of all EF components in PPA. This is recurrently reported as a limitation (Coemans et al., 2022) and diagnostic practice could benefit from a deeper understanding of PPA (variant) performance on this component. Different diagnostic criteria (Gorno-Tempini et al., 2011; Neary et al., 1998) were used to classify PPA variants within the studies of this systematic review as an unfortunate result of differences in publication dates, potentially affecting comparability across studies. However, although future studies should look at correlations between PPA subtype and executive dysfunctions, there remains to be clear merit in investigating the PPA population as a whole. Another limitation of our study is the inclusion of studies that used composite scores derived from multiple tasks, including tasks of less relevance to EF, which may reduce the ability to clearly link results to each specific executive function component. Moreover, though the review identified positive associations between EFs and neural correlates in PPA, these associations can only be interpreted as indicative. Additionally, in considering the patient groups, the three variants of PPA may show differing characteristics; however, not all included studies separated PPA variants in their analyses. Moreover, the studies included in our analysis did not provide pathological diagnoses for patients; the data provided were based primarily on clinical diagnoses. The functional neuroimaging studies on individuals with PPA are currently very few. This limits us from contemplating further on the functional markers using these techniques, however, as these kinds of functional neuroimaging studies are becoming more widespread, a future study using Coordinate-Based Meta-Analysis would be insightful. Lastly, another limitation of the study is the use of different control groups, including individuals with subjective memory complaints and normative data, which potentially affect the comparability across studies.

## 5 Conclusion

Though further research is still needed to assess underlying functional mechanisms related to PPA pathology, the present study suggests that reductions in functional connectivity may indicate deficits in the left hemisphere neural networks related to cognitive functions. Although neural differences are complex and difficult to pinpoint to cognitive mechanisms with certainty, the findings support the continued incorporation of all functional assessments of executive dysfunction for PPA patients. Additionally, with widely differencing definitions (or the lack thereof) across studies of what EFs encompass, there is a need for a consensus for EF functions and their assessments. Still, our study indirectly supports Miyake et al.'s (2000) assumption of the frontal regions and inferior frontal junctions relevance for executive control. Moreover, our study suggests that the FTLD spectrum is associated with dysfunction in this area.

## Data Availability

The original contributions presented in the study are included in the article/supplementary material, further inquiries can be directed to the corresponding author.
